# Elucidation of Codon Usage Signatures across the Domains of Life

**DOI:** 10.1093/molbev/msz124

**Published:** 2019-05-20

**Authors:** Eva Maria Novoa, Irwin Jungreis, Olivier Jaillon, Manolis Kellis

**Affiliations:** 1 Computer Science and Artificial Intelligence Laboratory, Massachusetts Institute of Technology, Cambridge, MA, USA; 2 Broad Institute of MIT and Harvard, Cambridge, MA, USA; 3 Garvan Institute of Medical Research, Darlinghurst, NSW, Australia; 4 University of New South Wales Sydney, NSW, Australia; 5 Génomique Métabolique, Genoscope, Institut François Jacob, CEA, CNRS, Univ Evry, Université Paris-Saclay, Evry, France

**Keywords:** codon usage, codon autocorrelation, tRNA, evolution

## Abstract

Because of the degeneracy of the genetic code, multiple codons are translated into the same amino acid. Despite being “synonymous,” these codons are not equally used. Selective pressures are thought to drive the choice among synonymous codons within a genome, while GC content, which is typically attributed to mutational drift, is the major determinant of variation across species. Here, we find that in addition to GC content, interspecies codon usage signatures can also be detected. More specifically, we show that a single amino acid, arginine, is the major contributor to codon usage bias differences across domains of life. We then exploit this finding and show that domain-specific codon bias signatures can be used to classify a given sequence into its corresponding domain of life with high accuracy. We then wondered whether the inclusion of codon usage codon autocorrelation patterns, which reflects the nonrandom distribution of codon occurrences throughout a transcript, might improve the classification performance of our algorithm. However, we find that autocorrelation patterns are not domain-specific, and surprisingly, are unrelated to tRNA reusage, in contrast to previous reports. Instead, our results suggest that codon autocorrelation patterns are a by-product of codon optimality throughout a sequence, where highly expressed genes display autocorrelated “optimal” codons, whereas lowly expressed genes display autocorrelated “nonoptimal” codons.

## Introduction

Despite the relative universality of the genetic code and the conservation of the translation machinery across species, synonymous codons are not equally used, and codon biases vary dramatically between organisms and across genes within the same genome (Hershberg and Petrov 2008; Plotkin and Kudla 2011; Novoa and Ribas de Pouplana 2012; Shabalina et al. 2013). Various factors can influence codon usage bias within and across genomes, including protein expression level (Gouy and Gautier 1982; Ikemura 1985), GC content (Hershberg and Petrov 2009; Palidwor et al. 2010), recombination rates (Marais et al. 2001), translation efficiency (Sorensen et al. 1989; Tuller, Waldman, et al. 2010; Qian et al. 2012), mRNA structure (Kudla et al. 2009), codon position (Tuller, Carmi, et al. 2010), mRNA stability (Presnyak et al. 2015), and gene length (Eyre-Walker 1996; Duret and Mouchiroud 1999), amongst others.

Although each species has a preference toward a specific subset of codons (Hershberg and Petrov 2009; Plotkin and Kudla 2011), the origin and evolutionary pressures driving these preferences remains largely unknown. Codon usage variation within genomes (intraspecies codon usage) is often attributed to selection, due to the significant positive correlation between protein expression levels and the presence of “preferred” or “optimal” codons (Sharp et al. 1986; Duret and Mouchiroud 1999). Indeed, both in Bacteria and Eukarya, codon bias is more extreme in highly expressed genes to match the skew of tRNA gene pools, providing a fitness advantage due to increased efficiency and/or accuracy in protein synthesis (Bulmer 1991; Akashi 1994; Dong et al. 1996; Duret 2000; Gingold and Pilpel 2011). In contrast, the processes that drive codon usage variation across genomes (interspecies codon usage) are generally thought to be mutational (Chen et al. 2004; Hershberg and Petrov 2008; Sharp et al. 2010), although the extent to which these processes are driven by mutation, selection, or biased gene conversion remains controversial (Lassalle et al. 2015; Long et al. 2018). Genomic GC content has been identified as the strongest determinant of codon usage variation across species (Knight et al. 2001; Palidwor et al. 2010). Consequently, GC-rich organisms tend to favor GC-rich codons whereas AT-rich organisms are enriched in AT-rich codons (Hershberg and Petrov 2009).

tRNA gene content and codon usage bias are thought to coevolve (Dong et al. 1996; Yona et al. 2013) as a means to modulate translation speed for accurate cotranslational protein folding (Komar 2009; Yu et al. 2015). Indeed, tRNA deletions in *Saccharomyces cerevisiae* are recurrently corrected, with the anticodon of a second tRNA mutated to match that of the deleted tRNA (Yona et al. 2013). Supporting this, species belonging to the same domain of life (Archaea, Bacteria, Eukarya) were found to have evolved similarly in terms of their tRNA gene contents and decoding strategies (Novoa et al. 2012), despite large differences in GC content. In the light of these observations, here we hypothesize and test whether species from the same domain of life may display similar codon usage biases despite their differences in GC content.

The elucidation of which evolutionary pressures have shaped extant genomes is crucial to comprehending why and how genomes evolve, but also can be exploited to build algorithms that can taxonomically annotate any given genomic sequence based on its properties. In this regard, next-generation sequencing has provided a great opportunity to explore complex ecological systems, such as microbiomes from the human gut or environmental samples. However, these samples often include a significant portion of uncharacterized species, and consequently, assigning sequence scaffolds to individual species, or even to higher-level taxa, remains challenging. Metagenomic annotation solutions, also known as “binning,” often rely on similarity-based searches (Brady and Salzberg 2009; Gerlach and Stoye 2011; Huson et al. 2016). Unfortunately, such homology-based methods are unable to correctly annotate a significant portion of sequences (Prakash and Taylor 2012), such as those that are taxonomically restricted, or do not have detectable homologues in other lineages. De novo taxonomical predictions, independent of homology, can be extremely useful for these situations.

Here, we show that after removing variation associated with GC content, species from the same domain share similar codon bias signature, and identify that the codon usage bias of a single amino acid, arginine, is largely responsible for the separation of the species into their corresponding domains. We then show that coding sequences (CDS) can be correctly classified into their corresponding domain of life, with an accuracy of 85%, using exclusively their codon usage biases. We speculate that domain-specific preferences for arginine codons are related to translation speed, which would support the view that codon usage variation across genomes is shaped not only by mutational biases, but also by selective forces.

## Results

### Beyond GC Content, Codon Usage Bias Shows Domain-Specific Patterns

**Fig. 1.**
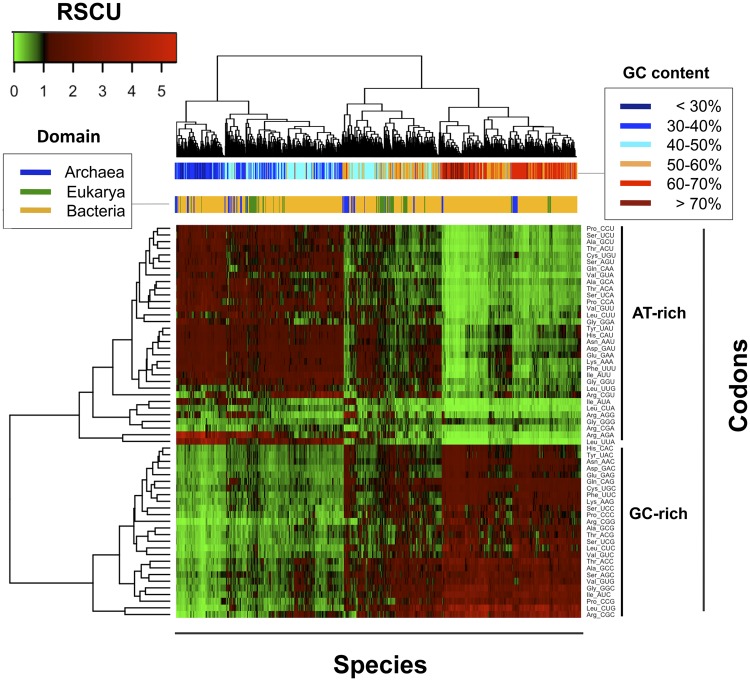
Analysis of codon usage bias across the three domains of life. (*A*) Hierarchical clustering of the average relative synonymous codon usage (RSCU) for each species (*n* = 1,625). Horizontal bars indicate GC content and domain of life for each species, and show that RSCU clusters species primarily by their GC content rather than by domain.

The nonuniform usage of synonymous codons in a given sequence or genome can be measured as relative synonymous codon usage (RSCU), which is defined as the ratio of the observed frequency of codons to the expected frequency. In other words, the RSCU represents the deviation of the observed codon usage from a uniform distribution in which all codons encoding for the same amino acid have the same probability (Sharp et al. 1986). Therefore, the codon usage bias of each species can be represented by a 59-dimensional RSCU vector, where each element of the vector is the RSCU of an individual codon (Trp, Met, and stop codons are excluded). Upon hierarchical clustering of species based on their average RSCU, we find that species do not cluster following the tree of life, but rather, based on GC content, suggesting GC content is the major determinant of codon usage bias across species (fig. 1), in agreement with previous works (Hershberg and Petrov 2009).

**Fig. 2.**
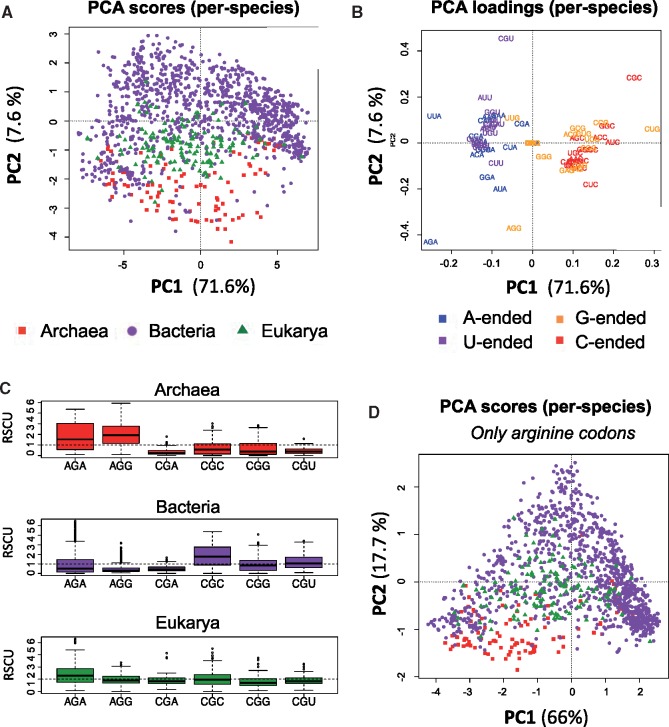
(*A*) Scatter plot of the first two principal components of the matrix of RSCU values in panel *A*. Each dot represents a species, and has been colored according to its corresponding domain. (*B*) PCA loadings plot for the first two principal components, where each codon has been colored according to its ending nucleotide: G (orange), C (red), A (blue), or T (purple), showing that the PC1 score of a species is primarily determined by differences in frequencies of codons ending in GC or AT, whereas PC2 is mainly driven by differences in frequencies of arginine codons more than those of any other amino acid. See also supplementary table S1, Supplementary Material online for individual contributions of codons to each PC. (*C*) Boxplot representation of arginine codon usage for each domain of life, showing that Archaea favor AGA and AGG codons, Bacteria favor CGC and CGU codons, and Eukarya show intermediate preferences. (*D*) 3D scatter plot representing each species by its first three principal component scores, using as input only the RSCU values of arginine codons, showing that arginine codon usage alone allows for discrimination of domains. See also supplementary figure S1, Supplementary Material online for additional principal components and supplementary table S1, Supplementary Material online for individual contributions of each codon to PC2.

To deconvolute the bias related to GC content from that caused by codon usage, we applied principal component analysis (PCA) to the average RSCU of each analyzed species (see Materials and Methods), with the expectation that the first principal component (PC1) would capture the variance due to GC content. Indeed, we find that PC1 does not separate the species into domains (fig. 2*A*, see also supplementary fig. S1, Supplementary Material online), but clusters them according to GC content, as reflected by the contributions of the individual codons to PC1 (i.e., GC-ended codons have negative PC1 scores, whereas AT-ended codons have positive PC1 scores; fig. 2*B*). In contrast, the second principal component (PC2) is capable of separating species into their corresponding domains of life (fig. 2*A*), confirming our hypothesis.

### Arginine Codons Are the Major Drivers of Interspecies Codon Usage Bias

Four codons, AGA, AGG, CGU, and CGC, all coding for arginine, are the largest contributors to the separation of species in PC2 (fig. 2*B*, see also supplementary table S1, Supplementary Material online for individual contributions of each codon to each PC). Upon closer examination of arginine codon usage biases in the three domains, we observe that their relative usage of arginine codons is distinct (fig. 2*C*). More specifically, Archaea preferentially use AGG and AGA, whereas Bacteria preferentially use CGC and CGU, and Eukarya show intermediate preferences between Archaea and Bacteria.

We then wondered whether the arginine codon bias was sufficient to cluster species into their corresponding domains. To test this, we performed a new PCA analysis, this time using exclusively arginine codon biases, and finding that the differences in arginine codon usage across species alone were sufficient to recapitulate the clustering of species into domains (fig. 2*D*). Therefore, we conclude that arginine codon usage bias is a major contributor to interspecies codon usage bias across domains.

### The Usage of Arginine Codons Does Not Significantly Change across Highly and Lowly Expressed Proteins

**Fig. 3.**
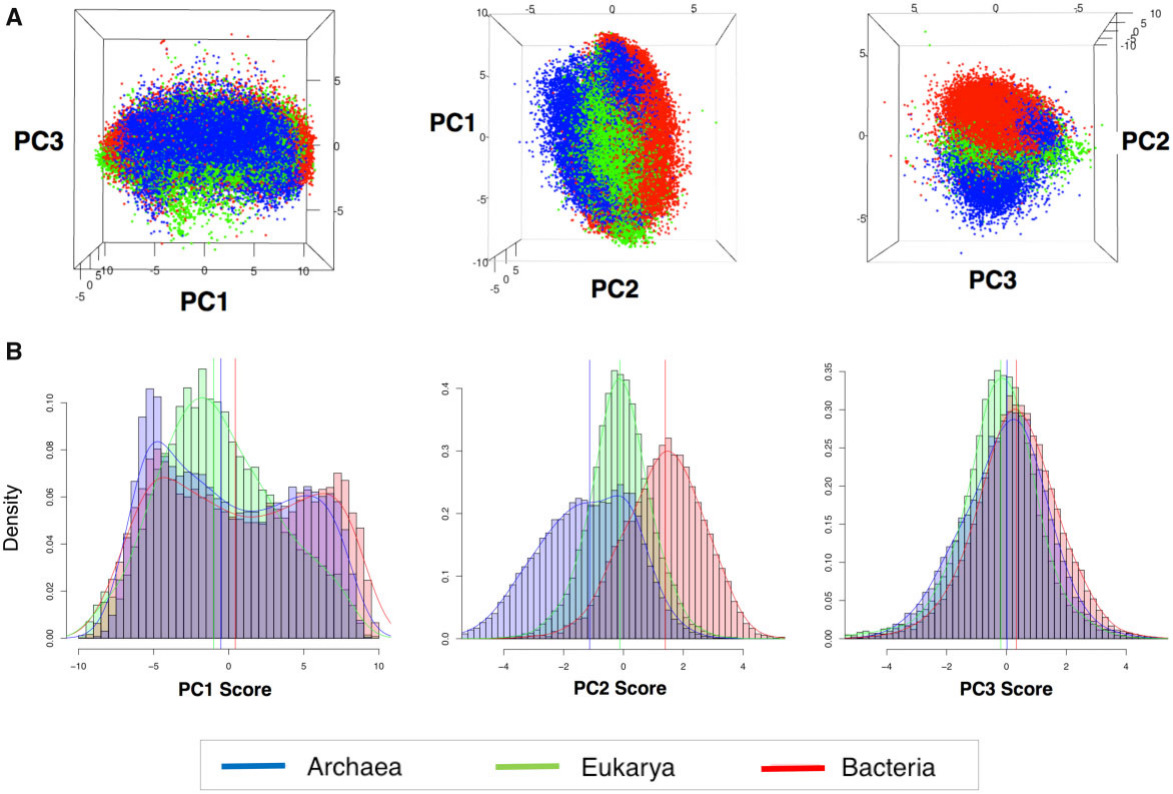
Codon usage bias clusters sequences into their corresponding domains. (*A*) 3D scatter plot of the first three principal component scores for all EMBLCDS sequences included in the analysis. Each dot represents a sequence, and has been colored by its corresponding domain of life: Archaea (blue), Bacteria (red), Eukarya (green). (*B*) Histograms of the densities of the PC scores for each domain: PC1 scores (left), PC2 scores (middle), PC3 scores (right).

Within a given species, the usage of individual codons across its genes is not uniform. Specifically, highly expressed proteins tend to be more enriched in “optimal” of “preferred” codons, compared with proteins with lower expression (Bulmer 1987; Duret 2000; Higgs and Ran 2008; McDonald et al. 2015). Therefore, considering this variation within-species, we wondered whether our findings would be applicable at the level of individual sequences. For this aim, we individually analyzed all sequences from the 1,625 EMBLCDS species from all three domains (see Materials and Methods). We computed RSCU values for each sequence, performed PCA dimensionality reduction, and retained the first three principal components for further analysis, based on Scree’s test (Cattell 1966). We found that individual sequences also clustered by domain, suggesting that intraspecies codon usage variation is not larger than interspecies codon usage variation (fig. 3).

**Fig. 4.**
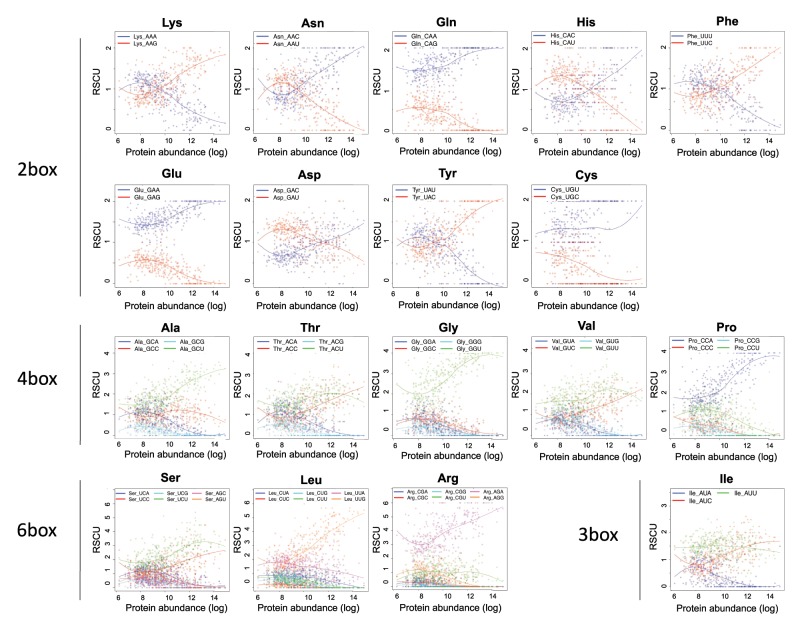
Codon preferences in *Saccharomyces cerevisiae* as a function of expression levels. Codon preferences, represented by relative synonymous codon usage (RSCU), are reversed between highly and lowly expressed genes for some amino acids but not for others. Although codon usage varies within a genome, intragenome differences are small enough that individual sequences still cluster by domain, as seen in figure 2. See also supplementary figure S2, Supplementary Material online for equivalent plots in *Escherichia coli*.

Surprised by these results, we hypothesized that although global codon usage may strongly vary between highly and lowly expressed genes (Bulmer 1987; Duret 2000; Higgs and Ran 2008; McDonald et al. 2015), this might not be the case for all amino acids, such as arginine. To test this, we examined the codon usage of all CDS of *S. cerevisiae*, and determined how codon usage varied with protein expression—using previously published proteomics data sets (Newman et al. 2006)—for each individual amino acid and codon subtype (fig. 4). As expected, we observed that codon usage drastically varied depending on protein abundance. For the majority of amino acids, codon preferences completely switch from “nonoptimal” to “optimal” depending on expression level (Lys, Asn, His, Phe, Asp, Tyr, Val, Ser, Ile)—here considering that a codon preference “switch” occurs if the most frequently used codon in lowly expressed proteins differs from the one that is most frequently used in highly expressed proteins. In other cases, however, codon preferences are maintained—although RSCU values may vary—when comparing lowly and highly expressed proteins (Gln, Glu, Cys, Gly, Arg). The relative consistency of arginine codon usage preferences across a genome might explain why our analyses can be applied not only at an average per-species level, but also at the level of individual sequences. Similar results were observed when the same analysis was performed on *Escherichia coli*, where codon preferences of certain amino acids switch from “nonoptimal” to “optimal” (His, Phe, Asp, Tyr, Ala, Gly, Val, Ser, Ile), whereas in others the same codon is preferentially used in both highly and lowly expressed proteins (Lys, Asn, Gln, Glu, Cys, Thr, Pro, Leu, Arg; supplementary fig. S2, Supplementary Material online).

### Codon Usage Signatures Can Be Used to Taxonomically Annotate Sequences into Their Corresponding Domains of Life

**Fig. 5.**
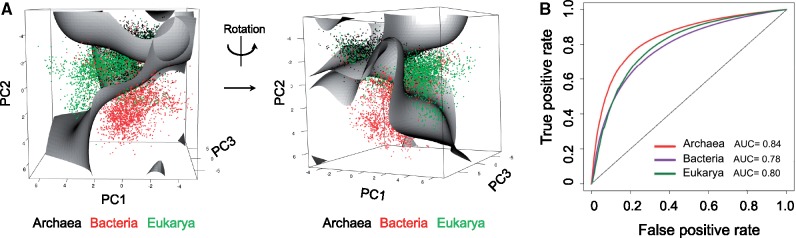
Taxonomical classification of sequences using codon usage bias. (*A*) 3D plot representation of the first three principal component scores. Support Vector Machine hyperplanes computed using the training set are also shown. Each dot represents a sequence, and has been colored according to its corresponding domain of life. (*B*) ROC curves of the SVM class probabilities, computed separately for each domain. See also supplementary figure S3, Supplementary Material online.

We then wondered whether simple patterns of codon usage bias would be sufficient to classify a species into its corresponding domain of life. To test this, we used the previously built 59-dimensional RSCU vectors for each EMBLCDS sequence, subdivided the data into training and testing sets, and built a Support Vector Machine (SVM) with the training set data (fig. 5*A*). We find that codon usage bias alone predicts the correct domain with AUC values ranging from 0.78 to 0.84 (fig. 5*B*). The accuracy of prediction is dependent on the sequence length as could be expected (supplementary fig. S3, Supplementary Material online), however, predictions were found to be better than random even when analyzing the shortest set of CDS sequences (100–200 nt).

We then investigated whether this approach could be used to classify species into their corresponding phylum, and not just domain of life. For this aim, we retrieved the corresponding phylum for each of the species included in the analysis using NCBI Taxonomy, and trained three new SVMs for archaeal, bacterial and eukaryal EMBLCDS sequences, respectively (supplementary table S2, Supplementary Material online). We find that our methodology can identify the correct phylum with an overall accuracy of 0.359 (Bacteria), 0.589 (Eukarya), and 0.901 (Archaea) (supplementary table S3, Supplementary Material online). Overall, we find that sequences belonging to specific phylums, such as Fusobacteria or Chlamydiae, can be classified with reasonable accuracy (0.84 and 0.90, for Fusobacteria and Chlamydiae, respectively); however, in the majority of the cases, codon usage bias seems to be insufficient to accurately classify a sequence at the phylum level (supplementary table S4, Supplementary Material online).

### Codon Autocorrelation Does Not Reflect tRNA Reuse

Previous studies in yeast have shown that once a particular codon has been used, subsequent occurrences of the same amino acid in the same transcript are not random (Cannarozzi et al. 2010), a phenomenon termed as “codon autocorrelation” or “codon covariation.” Mechanistically, it was argued that tRNA recycling was the driving force causing the observed biased distribution of synonymous codons along a sequence, that is, codons that would reuse the same tRNA would be favored as a means to increase the speed of translation (Cannarozzi et al. 2010). A subsequent study re-examined this question, and compared the autocorrelation between codons encoding the same amino acids to those encoding different ones (Hussmann and Press 2014). Intriguingly, this second study found that covariation between codons encoding different amino acids was as strong as covariation between codons encoding the same amino acid, concluding that there was insufficient evidence to claim that tRNA recycling is the force driving codon autocorrelation. Despite the uncertain cause of codon covariation, both studies show that the probability of observing a specific codon is dependent on previous codon occurrences, at least in the case of *S. cerevisiae*.

**Fig. 6.**
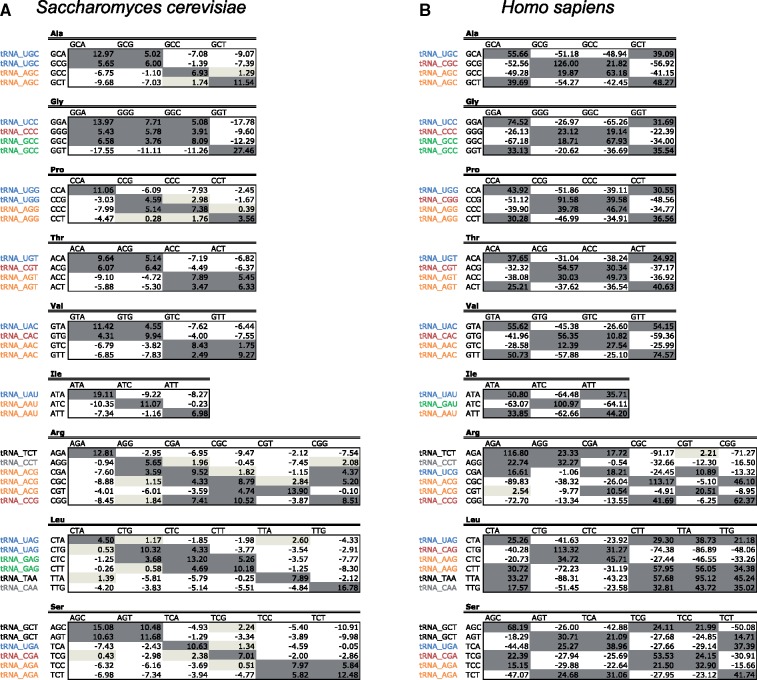
Analysis of codon covariation across species does not support a universal tRNA recycling model. Codon covariation measured over all pairs comprised of one codon and the subsequent one encoding for the same amino acid, shown for *Saccharomyces cerevisiae* (*A*) and *Homo sapiens* (*B*). Values correspond to standard deviations from expected. Each codon has been labeled with its corresponding decoding tRNA, following parsimony-extended wobble rules when no Watson-Crick matching tRNA isoacceptor is available (as per gtRNAdb, Chan and Lowe 2016). Pairs have been shaded according to the number of standard deviations from expected: dark gray (>+3SD; strongly favored codon pair), light gray (0–3SD; slightly favored codon pair), white (≤0 SD; nonfavored codon pair). In yeast, most codon pairs using the same tRNA are overrepresented, supporting a tRNA recycling model to explain the overrepresentation, but that is not true in other species. See also supplementary figure S4, Supplementary Material online for similar codon covariation analyses for *Escherichia coli* and *Plasmodium falciparum*.

Considering that species from the same domain of life share common codon usage signatures (figs. 2 and 3), we wondered whether codon covariation would also follow a similar behavior. For this aim, we calculated codon covariation as described in Cannarozzi et al. (2010) (see Materials and Methods), finding that codon covariation within same amino acids in *S. cerevisiae* partly supports a tRNA recycling model (fig. 6*A*). For example, in the case of alanine codons, GCA and GCG show covariation, and are both decoded by ${\text{tRNA}}_{\text{UGC}}^{\text{Ala}}$. Similarly, GCC and GCT are decoded by ${\text{tRNA}}_{\text{AGC}}^{\text{Ala}}$ and also show covariation.

In contrast, the covariation observed in other species was observed between codons that were decoded by different tRNAs (fig. 6*B*, see also supplementary fig. S4, Supplementary Material online). Taking as an example the same amino acid, alanine, covariation in human sequences was detected between GCA and GCT codons, which are decoded by two different tRNAs, ${\text{tRNA}}_{\text{UGC}}^{\text{Ala}}$ and ${\text{tRNA}}_{\text{AGC}}^{\text{Ala}}$, as well as between GCG and GCC codons, despite being decoded by two different tRNAs, ${\text{tRNA}}_{\text{CGC}}^{\text{Ala}}$ and ${\text{tRNA}}_{\text{AGC}}^{\text{Ala}}$ (fig. 6*B*). Similarly, in *E. coli*, the two alanine codons that show covariation are GCA and GCT, despite being decoded by two different tRNAs, ${\text{tRNA}}_{\text{UGC}}^{\text{Ala}}$ and ${\text{tRNA}}_{\text{GGC}}^{\text{Ala}}$ (supplementary fig. S4, Supplementary Material online). Overall, our results suggest that codon covariation is unrelated to tRNA reusage, in agreement with the second study described above (Hussmann and Press 2014).

### Codon Autocorrelation Reflects Global Sequence Codon Optimality

**Fig. 7.**
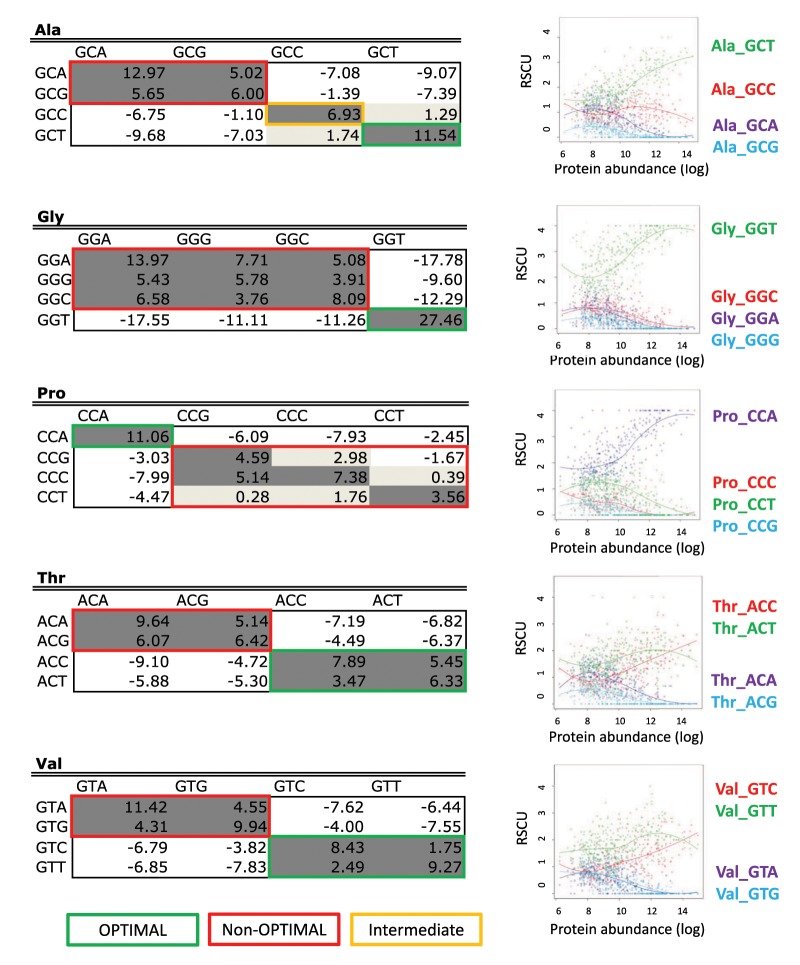
Codon covariation is likely a consequence of the co-occurrence of “optimal” and “nonoptimal” codons, in highly and lowly expressed proteins, respectively. Codon covariation for *Saccharomyces cerevisiae* as depicted in figure 6, highlighting those pairs that are formed by two optimal codons (dark green), two nonoptimal codons (red), and codons with intermediate optimality (yellow). Optimal and nonoptimal codons have been defined as those that are highly abundant and lowly abundant in highly expressed proteins, and their relative abundance is shown for each individual amino acid and codon. See also supplementary figure S5, Supplementary Material online for the analysis for all amino acids *S. cerevisiae*, as well as for same analysis performed in *Escherichia coli*.

We then wondered whether codon covariations may be in fact a simple consequence of codon optimality throughout a sequence, that is, whether “optimal” codons, which are abundant in highly expressed proteins, would appear as autocorrelated, and “nonoptimal” codons, which are abundant in lowly expressed proteins, would also appear as autocorrelated. To test this, we compared the observed codon covariations in *S. cerevisiae* and *E. coli* with the set of “optimal” and “nonoptimal” codons, defined as those that were highly or lowly abundant in highly expressed proteins, respectively (fig. 7). We find that after binning codons into “optimal” and “nonoptimal,” codon covariations were present within “optimal” or within “nonoptimal” codons, but not across them. More specifically, 97% (31/32) of the autocorrelated codon pairs (SD ≥ 3) in *S. cerevisiae* (fig. 7) and 86% (25/29) of the autocorrelated pairs (SD ≥ 3) in *E. coli* (supplementary fig. S5, Supplementary Material online) could be explained by codon optimality. It is important to note that the remaining autocorrelated codon pairs in *E. coli* (4/29) and in *S. cerevisiae* (1/32) actually correspond to codons which we labeled as “intermediate optimal” (yellow boxes), for which we considered that we could not clearly assign the category of “optimal” or “nonoptimal,” and thus were not counted as positive results. Overall, our results suggest that, at least in the case of *S. cerevisiae* and *E. coli*, codon autocorrelation may be a consequence of similar choice of “optimal” or “nonoptimal” codons throughout a sequence.

### Codon Autocorrelation Is Not Domain-Specific

Regardless of the evolutionary forces driving codon covariation, our analysis demonstrates that codon covariation exists in all species analyzed (fig. 6 and supplementary fig. S4, Supplementary Material online). Therefore, we extended this analysis to hundreds of species across the three domains of life (see Materials and Methods), and examined whether taxonomically related species displayed similar codon covariation patterns. We found that the number of standard deviations from the expected codon pair usage (Cannarozzi et al. 2010) was not a useful metric to compare species, due to dependence of this metric on genome size (fig. 6 and supplementary fig. S4, Supplementary Material online). Therefore, we defined a new metric, independent of genome size, which we termed Relative Synonymous Codon Pair Usage (RSCPU; see Materials and Methods). This metric represents the ratio of the observed usage of a given codon pair to the expected pair usage, which is defined as the product of the observed usage of the two individual codons in the genome. It is important to note that RSCPU values are normalized by the individual usage of the two codons in the genome, and thus are independent of GC content.

**Fig. 8.**
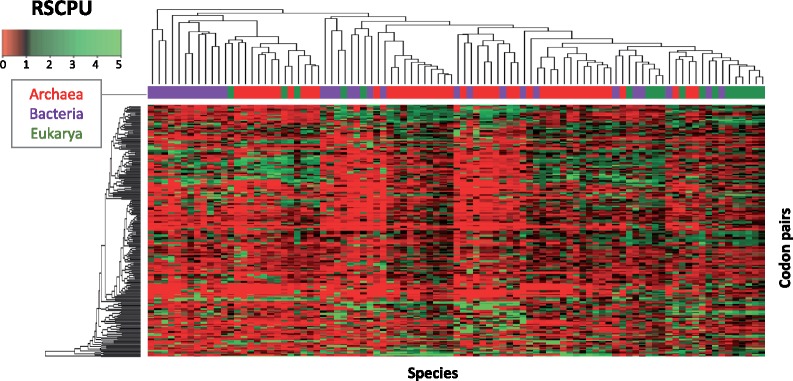
Hierarchical clustering of codon covariation patterns across species spanning the three domains of life. Each codon pair has been colored according to its RSCPU value. The upper bar over the heatmap represents the corresponding domain of each species. See also supplementary figure S6, Supplementary Material online.

Our results show that taxonomically related species display similar codon variation, however, multiple clusters appear within each domain of life, suggesting that codon covariation is not domain-specific (fig. 8). Nevertheless, we do observe that some species belonging to the same domain cluster together, suggesting that codon covariation is not completely independent of their taxonomy. We then performed PCA analysis on the RSCPU values to test whether additional principal components might better separate the species into their corresponding domains; however, we find that species belonging to different domains largely overlap (supplementary fig. S6, Supplementary Material online). Overall, our results suggest that codon covariation patterns are not domain-specific, but they do show certain degree of clustering which is dependent on their taxonomy. Therefore, we suggest that codon covariation signatures may be used as additional features to improve the performance of current binning algorithms, but cannot be used alone to classify species into their corresponding domains of life.

## Discussion

It is well established that the identity of favored codons varies among organisms (Chen et al. 2004; Hershberg and Petrov 2009; Sharp et al. 2010). However, the rules governing the identities of favored codons across organisms still remain obscure. High GC content organisms tend to have GC-rich favored codons, whereas low GC content organisms favor AT-rich codons, suggesting evolutionary pressures act in the same direction as the nucleotide substitution biases that determine overall nucleotide content of genomes (Hershberg and Petrov 2009). On the basis of these observations, previous works suggested that interspecies codon bias is driven mostly by genome-wide mutational biases. Here we suggest that, in addition to mutational forces, interspecies codon bias might also be shaped by selective forces.

Several studies have reported codon translation rates for a wide variety of species and conditions (Li et al. 2012; Gardin et al. 2014; Lareau et al. 2014; Bazzini et al. 2016). In a scenario of high abundance of nutrients, arginine codons have been recurrently identified as the slowest translated codons, both in Bacteria (Bonekamp and Jensen 1988; Chevance et al. 2014) and Eukarya (Charneski and Hurst 2013; Gardin et al. 2014; Requiao et al. 2016). However, within a species, not all arginine codons display slow translation rates. For example, in a bacterial system based on *Salmonella enterica*, CGC codons are rapidly translated, whereas AGG codons, which also encode for arginine, are slowly translated (Chevance et al. 2014). In the eukaryote *S. cerevisiae*, codons that are rapidly and slowly translated differ from those identified in bacteria. More specifically, CGC is slowly translated in *S. cerevisiae*, whilst AGA and CGU are rapidly translated (Gardin et al. 2014), matching the codon preferences of *S. cerevisiae* (fig. 4). Moreover, in the case of Bacteria, the variance in translation speed between codons that encode for arginine is larger than for any other amino acid, and it is also high in the case of Eukarya (supplementary fig. S7*A*, Supplementary Material online). In the light of these observations, we suggest that selective pressures toward maintaining specific arginine codon usage biases, compared with other amino acids, might be responsible for the existence of domain-specific arginine codon usage biases.

A remaining question, however, is why such extreme variance in translation speed of arginine codons exists. It has been suggested that the rare usage and slow translation speed of AGG codons in Bacteria is a consequence of their similarity to Shine-Dalgarno sequences (Li et al. 2012; Chevance et al. 2014; supplementary fig. S7*B*, Supplementary Material online). In agreement with this hypothesis, Shine-Dalgarno-like sequences are typically depleted in bacterial genomes (Li et al. 2012). However, Shine-Dalgarno sequences are also employed by archaeal genomes to help recruit the ribosome to initiate protein synthesis, and in this domain, AGG codons—together with AGA codons—are in fact the most frequently used codons. Therefore, it is unlikely that the similarity to Shine-Dalgarno sequences alone is responsible for the depletion of AGG codons in Bacteria. An alternate—but not mutually exclusive—explanation for this phenomenon might reside in the differences between arginine tRNA decoding strategies used in Archaea and Bacteria (Novoa et al. 2012). More specifically, in Bacteria, the appearance of tRNA adenosine deaminases (*tadA*), which allows for efficient translation of CGC and CGU codons, might explain why these two codons are more frequently used and rapidly translated in Bacteria (supplementary fig. S7*C*, Supplementary Material online). Moreover, *tadA* does not exist in Archaea, and thus it may explain why archaeal genomes do not preferentially use CGN codons. Future work will be needed to decipher which are the forces causing distinct arginine codon usage bias across different domains of life.

Regardless of the evolutionary forces driving domain-specific codon preferences, here we find that codon usage bias of individual sequences can be used to taxonomically annotate them at the domain level (fig. 5), largely due to differences in arginine codon usage (fig. 2 and supplementary table S1, Supplementary Material online). To improve the performance of the algorithm, we wondered whether we could also include codon covariation, which is a well-documented phenomenon in yeast (Cannarozzi et al. 2010; Hussmann and Press 2014). We find complex covariations of codon pairs are present in all analyzed species (fig. 6 and supplementary fig. S4, Supplementary Material online), which do not seem to be related to tRNA recycling, but rather, to similar codon optimality throughout a sequence (fig. 7 and supplementary fig. S5, Supplementary Material online). Unfortunately, codon covariation patterns were not shared by the species within each domain of life (fig. 8 and supplementary fig. S6, Supplementary Material online).

Composition-based binning methods have been previously applied to taxonomically annotate metagenomic sequences (McHardy et al. 2007; Brady and Salzberg 2009; Diaz et al. 2009; Rosen et al. 2011; Alneberg et al. 2014; Lin and Liao 2016; Lu et al. 2017). These methods exploit the uniqueness of base composition—from single to oligonucleotide levels—found across the genomes of different taxonomic entities, and have been implemented in tools such as PhyloPhythia (McHardy et al. 2007), TETRA (Teeling et al. 2004), and TACOA (Diaz et al. 2009), amongst many others. However, the underlying evolutionary principles governing the observed k-mer variation across species remains largely unknown. Consequently, it may be difficult to improve such algorithms without a working hypothesis of which additional variables might affect performance. Here, we find that taxonomically related species display common covariation patterns, although these patterns are not domain-specific. In the light of these findings, we propose that in addition to considering features such as overall k-mer counts (Teeling et al. 2004; Lu et al. 2017), codon covariation may be used to further improve the performance of current composition-based metagenomics binning algorithms.

## Materials and Methods

### Gene Sequences

The full set of EMBL CDS sequences was downloaded in July 2015 from ftp://ftp.ebi.ac.uk/. Species were clustered by its corresponding species and strain or subspecies (when available). To avoid overrepresented subsets of sequences (e.g., housekeeping genes from organisms for which only few sequences are included in the data set), species for which only few sequences were available were discarded. More specifically, species for which there were >1,000 genes in the EMBL CDS data set for that same species were selected for further analysis. The final set of EMBL CDS sequences analyzed consisted in 1,625 genomes, which included over 14 million sequences. The distribution of sequences per phylum can be found in supplementary table S2, Supplementary Material online.

### Training and Test Set Preparation

The filtered set of EMBL CDS sequences was divided into training (10%) and test set (90%). Each training set sequence was individually analyzed in terms of codon usage bias, and converted into a 59-element vector (one for each codon, excluding Met, Trp, and stop codons) of RSCU values. RSCU was computed as defined in Sharp et al. (1986). PCA was applied on the training set matrix of RSCU values to reduce the dimensionality of the data. Scree’s test (Cattell 1966) was used to determine the number of significant principal components to be retained. PCA scores of the selected subset of principal components were used as new vectors to define each sequence. Each sequence was assigned to a domain (Eukarya, Bacteria, Archaea) based on its taxonomical annotation in NCBI taxonomy.

### Machine Learning

PCA scores of the EMBL CDS training set with its corresponding domain annotations were used as input to train a SVM using the e1071 library from R, using a C-classification method and a class.weights vector to compensate for asymmetric class sizes. Parameters were optimized using the tune.svm function. The final SVM model was validated using 5-fold cross-validation. EMBL CDS test set sequences were converted into PCA scores by applying the same PCA loadings that were generated upon PC analysis on the training set, and its corresponding domains were predicted using the SVM model built on the training set. Our model correctly predicted the domain of the EMBL CDS test set sequences with an overall accuracy of 85%.

### Codon Autocorrelation

Codon autocorrelation standard deviations were computed as described in Cannarozzi et al. (2010). Briefly, for each sequence, the number of consecutive pairs of codons for a same amino acid were counted. The expected numbers of pairs were computed as the products of the frequencies of the individual codons. Codons were Z-transformed by subtracting the expected counts from the observed and divided by the standard deviations from the expected value. The results were expressed as standard deviations from the expected value. Code for computing codon autocorrelation can be found in https://github.com/enovoa/codonAutocorrelation.

### RSCPU

We define the RSCPU as the ratio of the observed frequency (*f*_obs_pair_) of a given codon pair to the expected frequency of the codon pair (*f*_exp_pair_) (eq. 1). The expected frequency of the codon pair is defined as the product of the individual codon frequencies (*f*_obs_codon1_ and *f*_obs_codon2_) observed in the genome (eq. 2).

(eq. 1) $$\text{RSCPU}\;=\;f_{\text{obs}\_\text{pair}}/f_{\;\text{exp}\_\text{pair}}$$

(eq. 2) $$f_{\;\text{exp}\_\text{pair}}\;=\;f_{\text{obs}\_\text{codon}1}\;*\;f_{\text{obs}\_\text{codon}2}$$

Therefore, if the observed frequency matches the expected frequency, the RSCPU will have a value of 1. If the RSCPU is higher than 1, the pair is seen more frequently than what would be expected considering the individual codon frequencies, whereas if it is lower than 1, the pair is observed less frequently than what would be expected.

### Protein Abundances

Protein abundance values of *S. cerevisiae*, used to build figures 4 and 7, were taken from the work of Newman et al. (2006). Protein abundance values of *E. coli*, used to build supplementary figure S2, Supplementary Material online, were taken from Lu et al. (2007). The fitting of RSCU values relative to protein abundances (log) has been done using the *loess* function in R, which uses Local Polynomial Regression fitting.

### Code Availability

Code used in this work, including processed data, is freely available on github https://github.com/enovoa/codonAutocorrelation and https://github.com/enovoa/codonUsageToolkit.

## Supplementary Material

Supplementary data are available at *Molecular Biology and Evolution* online.

## Supplementary Material

msz124_Supplementary_Data

## References

[msz124-B1] Akashi H. 1994 Synonymous codon usage in *Drosophila melanogaster*: natural selection and translational accuracy. Genetics 136 3:927–935. 8005445 10.1093/genetics/136.3.927 PMC1205897

[msz124-B2] Alneberg J, Bjarnason BS, de Bruijn I, Schirmer M, Quick J, Ijaz UZ, Lahti L, Loman NJ, Andersson AF, Quince C. 2014 Binning metagenomic contigs by coverage and composition. Nat Methods. 11 11:1144–1146. 25218180 10.1038/nmeth.3103

[msz124-B3] Bazzini AA, Del Viso F, Moreno-Mateos MA, Johnstone TG, Vejnar CE, Qin Y, Yao J, Khokha MK, Giraldez AJ. 2016 Codon identity regulates mRNA stability and translation efficiency during the maternal-to-zygotic transition. EMBO J. 35 19:2087–2103. 27436874 10.15252/embj.201694699 PMC5048347

[msz124-B4] Bonekamp F, Jensen KF. 1988 The AGG codon is translated slowly in *E. coli* even at very low expression levels. Nucleic Acids Res. 16 7:3013–3024. 3285325 10.1093/nar/16.7.3013 PMC336448

[msz124-B5] Brady A, Salzberg SL. 2009 Phymm and PhymmBL: metagenomic phylogenetic classification with interpolated Markov models. Nat Methods 6 9:673–676. 19648916 10.1038/nmeth.1358 PMC2762791

[msz124-B6] Bulmer M. 1987 Coevolution of codon usage and transfer RNA abundance. Nature 325 6106:728–730. 2434856 10.1038/325728a0

[msz124-B7] Bulmer M. 1991 The selection-mutation-drift theory of synonymous codon usage. Genetics 129 3:897–907. 1752426 10.1093/genetics/129.3.897 PMC1204756

[msz124-B8] Cannarozzi G, Cannarrozzi G, Schraudolph NN, Faty M, von Rohr P, Friberg MT, Roth AC, Gonnet P, Gonnet G, Barral Y. 2010 A role for codon order in translation dynamics. Cell 141 2:355–367. 20403329 10.1016/j.cell.2010.02.036

[msz124-B9] Cattell RB. 1966 The scree test for the number of factors. Multivariate Behav Res. 1 2:245–276. 26828106 10.1207/s15327906mbr0102_10

[msz124-B10] Chan PP, Lowe TM. 2016 GtRNAdb 2.0: an expanded database of transfer RNA genes identified in complete and draft genomes. Nucleic Acids Res. 44(D1):D184–D189. 26673694 10.1093/nar/gkv1309 PMC4702915

[msz124-B11] Charneski CA, Hurst LD. 2013 Positively charged residues are the major determinants of ribosomal velocity. PLoS Biol. 11 3:e1001508. 23554576 10.1371/journal.pbio.1001508 PMC3595205

[msz124-B12] Chen SL, Lee W, Hottes AK, Shapiro L, McAdams HH. 2004 Codon usage between genomes is constrained by genome-wide mutational processes. Proc Natl Acad Sci U S A. 101 10:3480–3485. 14990797 10.1073/pnas.0307827100 PMC373487

[msz124-B13] Chevance FF, Le Guyon S, Hughes KT. 2014 The effects of codon context on in vivo translation speed. PLoS Genet. 10 6:e1004392. 24901308 10.1371/journal.pgen.1004392 PMC4046918

[msz124-B14] Diaz NN, Krause L, Goesmann A, Niehaus K, Nattkemper TW. 2009 TACOA: taxonomic classification of environmental genomic fragments using a kernelized nearest neighbor approach. BMC Bioinformatics 10:56. 19210774 10.1186/1471-2105-10-56 PMC2653487

[msz124-B15] Dong H, Nilsson L, Kurland CG. 1996 Co-variation of tRNA abundance and codon usage in *Escherichia coli* at different growth rates. J Mol Biol. 260 5:649–663. 8709146 10.1006/jmbi.1996.0428

[msz124-B16] Duret L. 2000 tRNA gene number and codon usage in the C. elegans genome are co-adapted for optimal translation of highly expressed genes. Trends Genet. 16 7:287–289. 10858656 10.1016/s0168-9525(00)02041-2

[msz124-B17] Duret L, Mouchiroud D. 1999 Expression pattern and, surprisingly, gene length shape codon usage in Caenorhabditis, Drosophila, and Arabidopsis. Proc Natl Acad Sci U S A. 96 8:4482–4487. 10200288 10.1073/pnas.96.8.4482 PMC16358

[msz124-B18] Eyre-Walker A. 1996 Synonymous codon bias is related to gene length in *Escherichia coli*: selection for translational accuracy? Mol Biol Evol. 13 6:864–872. 8754221 10.1093/oxfordjournals.molbev.a025646

[msz124-B19] Gardin J, Yeasmin R, Yurovsky A, Cai Y, Skiena S, Futcher B. 2014 Measurement of average decoding rates of the 61 sense codons in vivo. Elife 3. 10.7554/eLife.03735 PMC4371865 25347064

[msz124-B20] Gerlach W, Stoye J. 2011 Taxonomic classification of metagenomic shotgun sequences with CARMA3. Nucleic Acids Res. 39 14:e91. 21586583 10.1093/nar/gkr225 PMC3152360

[msz124-B21] Gingold H, Pilpel Y. 2011 Determinants of translation efficiency and accuracy. Mol Syst Biol. 7:481. 21487400 10.1038/msb.2011.14 PMC3101949

[msz124-B22] Gouy M, Gautier C. 1982 Codon usage in bacteria: correlation with gene expressivity. Nucleic Acids Res. 10 22:7055–7074. 6760125 10.1093/nar/10.22.7055 PMC326988

[msz124-B23] Hershberg R, Petrov DA. 2009 General rules for optimal codon choice. PLoS Genet. 5 7:e1000556. 19593368 10.1371/journal.pgen.1000556 PMC2700274

[msz124-B24] Hershberg R, Petrov DA. 2008 Selection on codon bias. Annu Rev Genet. 42:287–299. 18983258 10.1146/annurev.genet.42.110807.091442

[msz124-B25] Higgs PG, Ran W. 2008 Coevolution of codon usage and tRNA genes leads to alternative stable states of biased codon usage. Mol Biol Evol. 25 11:2279–2291. 18687657 10.1093/molbev/msn173

[msz124-B26] Huson DH, Beier S, Flade I, Gorska A, El-Hadidi M, Mitra S, Ruscheweyh HJ, Tappu R. 2016 MEGAN community edition – interactive exploration and analysis of large-scale microbiome sequencing data. PLoS Comput Biol. 12 6:e1004957. 27327495 10.1371/journal.pcbi.1004957 PMC4915700

[msz124-B27] Hussmann JA, Press WH. 2014 Local correlations in codon preferences do not support a model of tRNA recycling. Cell Rep. 8 6:1624–1629. 25199837 10.1016/j.celrep.2014.08.012

[msz124-B28] Ikemura T. 1985 Codon usage and tRNA content in unicellular and multicellular organisms. Mol Biol Evol. 2 1:13–34. 3916708 10.1093/oxfordjournals.molbev.a040335

[msz124-B29] Knight RD, Freeland SJ, Landweber LF. 2001 A simple model based on mutation and selection explains trends in codon and amino-acid usage and GC composition within and across genomes. Genome Biol. 2 4:RESEARCH0010. 11305938 10.1186/gb-2001-2-4-research0010 PMC31479

[msz124-B30] Komar AA. 2009 A pause for thought along the co-translational folding pathway. Trends Biochem Sci. 34 1:16–24. 18996013 10.1016/j.tibs.2008.10.002

[msz124-B31] Kudla G, Murray AW, Tollervey D, Plotkin JB. 2009 Coding-sequence determinants of gene expression in *Escherichia coli*. Science 324 5924:255–258. 19359587 10.1126/science.1170160 PMC3902468

[msz124-B32] Lareau LF, Hite DH, Hogan GJ, Brown PO. 2014 Distinct stages of the translation elongation cycle revealed by sequencing ribosome-protected mRNA fragments. Elife 3:e01257. 24842990 10.7554/eLife.01257 PMC4052883

[msz124-B33] Lassalle F, Perian S, Bataillon T, Nesme X, Duret L, Daubin V. 2015 GC-Content evolution in bacterial genomes: the biased gene conversion hypothesis expands. PLoS Genet. 11 2:e1004941. 25659072 10.1371/journal.pgen.1004941 PMC4450053

[msz124-B34] Li GW, Oh E, Weissman JS. 2012 The anti-Shine-Dalgarno sequence drives translational pausing and codon choice in bacteria. Nature 484 7395:538–541. 22456704 10.1038/nature10965 PMC3338875

[msz124-B35] Lin HH, Liao YC. 2016 Accurate binning of metagenomic contigs via automated clustering sequences using information of genomic signatures and marker genes. Sci Rep. 6:24175. 27067514 10.1038/srep24175 PMC4828714

[msz124-B36] Long H, Sung W, Kucukyildirim S, Williams E, Miller SF, Guo W, Patterson C, Gregory C, Strauss C, Stone C, et al 2018 Evolutionary determinants of genome-wide nucleotide composition. Nat Ecol Evol. 2 2:237–240. 29292397 10.1038/s41559-017-0425-y PMC6855595

[msz124-B37] Lu P, Vogel C, Wang R, Yao X, Marcotte EM. 2007 Absolute protein expression profiling estimates the relative contributions of transcriptional and translational regulation. Nat Biotechnol. 25 1:117–124. 17187058 10.1038/nbt1270

[msz124-B38] Lu YY, Chen T, Fuhrman JA, Sun F. 2017 COCACOLA: binning metagenomic contigs using sequence COmposition, read CoverAge, CO-alignment and paired-end read LinkAge. Bioinformatics 33 6:791–798. 27256312 10.1093/bioinformatics/btw290

[msz124-B39] Marais G, Mouchiroud D, Duret L. 2001 Does recombination improve selection on codon usage? Lessons from nematode and fly complete genomes. Proc Natl Acad Sci U S A. 98 10:5688–5692. 11320215 10.1073/pnas.091427698 PMC33274

[msz124-B40] McDonald MJ, Chou CH, Swamy KB, Huang HD, Leu JY. 2015 The evolutionary dynamics of tRNA-gene copy number and codon-use in *E. coli*. BMC Evol Biol. 15:163. 26282127 10.1186/s12862-015-0441-y PMC4539685

[msz124-B41] McHardy AC, Martin HG, Tsirigos A, Hugenholtz P, Rigoutsos I. 2007 Accurate phylogenetic classification of variable-length DNA fragments. Nat Methods. 4 1:63–72. 17179938 10.1038/nmeth976

[msz124-B42] Newman JR, Ghaemmaghami S, Ihmels J, Breslow DK, Noble M, DeRisi JL, Weissman JS. 2006 Single-cell proteomic analysis of *S. cerevisiae* reveals the architecture of biological noise. Nature 441 7095:840–846. 16699522 10.1038/nature04785

[msz124-B43] Novoa EM, Pavon-Eternod M, Pan T, Ribas de Pouplana L. 2012 A role for tRNA modifications in genome structure and codon usage. Cell 149 1:202–213. 22464330 10.1016/j.cell.2012.01.050

[msz124-B44] Novoa EM, Ribas de Pouplana L. 2012 Speeding with control: codon usage, tRNAs, and ribosomes. Trends Genet. 28 11:574–581. 22921354 10.1016/j.tig.2012.07.006

[msz124-B45] Palidwor GA, Perkins TJ, Xia X. 2010 A general model of codon bias due to GC mutational bias. PLoS One 5 10:e13431. 21048949 10.1371/journal.pone.0013431 PMC2965080

[msz124-B46] Plotkin JB, Kudla G. 2011 Synonymous but not the same: the causes and consequences of codon bias. Nat Rev Genet. 12 1:32–42. 21102527 10.1038/nrg2899 PMC3074964

[msz124-B47] Prakash T, Taylor TD. 2012 Functional assignment of metagenomic data: challenges and applications. Brief Bioinformatics 13 6:711–727. 22772835 10.1093/bib/bbs033 PMC3504928

[msz124-B48] Presnyak V, Alhusaini N, Chen YH, Martin S, Morris N, Kline N, Olson S, Weinberg D, Baker KE, Graveley BR, et al 2015 Codon optimality is a major determinant of mRNA stability. Cell 160 6:1111–1124. 25768907 10.1016/j.cell.2015.02.029 PMC4359748

[msz124-B49] Qian W, Yang JR, Pearson NM, Maclean C, Zhang J. 2012 Balanced codon usage optimizes eukaryotic translational efficiency. PLoS Genet. 8 3:e1002603. 22479199 10.1371/journal.pgen.1002603 PMC3315465

[msz124-B50] Requiao RD, de Souza HJ, Rossetto S, Domitrovic T, Palhano FL. 2016 Increased ribosome density associated to positively charged residues is evident in ribosome profiling experiments performed in the absence of translation inhibitors. RNA Biol. 13 6:561–568. 27064519 10.1080/15476286.2016.1172755 PMC4962802

[msz124-B51] Rosen GL, Reichenberger ER, Rosenfeld AM. 2011 NBC: the Naive Bayes Classification tool webserver for taxonomic classification of metagenomic reads. Bioinformatics 27 1:127–129. 21062764 10.1093/bioinformatics/btq619 PMC3008645

[msz124-B52] Shabalina SA, Spiridonov NA, Kashina A. 2013 Sounds of silence: synonymous nucleotides as a key to biological regulation and complexity. Nucleic Acids Res. 41 4:2073–2094. 23293005 10.1093/nar/gks1205 PMC3575835

[msz124-B53] Sharp PM, Emery LR, Zeng K. 2010 Forces that influence the evolution of codon bias. Philos Trans R Soc Lond B Biol Sci. 365 1544:1203–1212. 20308095 10.1098/rstb.2009.0305 PMC2871821

[msz124-B54] Sharp PM, Tuohy TM, Mosurski KR. 1986 Codon usage in yeast: cluster analysis clearly differentiates highly and lowly expressed genes. Nucleic Acids Res. 14 13:5125–5143. 3526280 10.1093/nar/14.13.5125 PMC311530

[msz124-B55] Sorensen MA, Kurland CG, Pedersen S. 1989 Codon usage determines translation rate in *Escherichia coli*. J Mol Biol. 207 2:365–377. 2474074 10.1016/0022-2836(89)90260-x

[msz124-B56] Teeling H, Waldmann J, Lombardot T, Bauer M, Glockner FO. 2004 TETRA: a web-service and a stand-alone program for the analysis and comparison of tetranucleotide usage patterns in DNA sequences. BMC Bioinformatics 5 1:163. 15507136 10.1186/1471-2105-5-163 PMC529438

[msz124-B57] Tuller T, Carmi A, Vestsigian K, Navon S, Dorfan Y, Zaborske J, Pan T, Dahan O, Furman I, Pilpel Y. 2010 An evolutionarily conserved mechanism for controlling the efficiency of protein translation. Cell 141 2:344–354. 20403328 10.1016/j.cell.2010.03.031

[msz124-B58] Tuller T, Waldman YY, Kupiec M, Ruppin E. 2010 Translation efficiency is determined by both codon bias and folding energy. Proc Natl Acad Sci U S A. 107 8:3645–3650. 20133581 10.1073/pnas.0909910107 PMC2840511

[msz124-B59] Yona AH, Bloom-Ackermann Z, Frumkin I, Hanson-Smith V, Charpak-Amikam Y, Feng Q, Boeke JD, Dahan O, Pilpel Y. 2013 tRNA genes rapidly change in evolution to meet novel translational demands. Elife 2:e01339. 24363105 10.7554/eLife.01339 PMC3868979

[msz124-B60] Yu CH, Dang Y, Zhou Z, Wu C, Zhao F, Sachs MS, Liu Y. 2015 Codon usage influences the local rate of translation elongation to regulate co-translational protein folding. Mol Cell. 59 5:744–754. 26321254 10.1016/j.molcel.2015.07.018 PMC4561030

