# Supplementary material for: Elucidation of Codon Usage Signatures across the Domains of Life
Source: Mol Biol Evol. 2019 May 20;36(10):2328–39. doi: 10.1093/molbev/msz124 (PMC6759073; doi:10.1093/molbev/msz124)
Supplement: msz124_Supplementary_Data [file msz124_supplementary_data.docx]

**Supplementary Information**

Elucidation of Codon Usage Signatures across the Domains of Life

Eva Maria Novoa^1,2,3,4*^, Irwin Jungreis^1,2^ , Olivier Jaillon^1,2,5^ and Manolis Kellis^1,2*^

*^1^Computer Science and Artificial Intelligence Lab, MIT, Cambridge 02139, MA, USA.*

*^2^Broad Institute of MIT and Harvard, Cambridge 02139, MA, USA.*

*^3^Garvan Institute of Medical Research, Darlinghurst 2010 NSW, Australia.*

*^4^University of New South Wales Sydney, Sydney NSW Australia.*

*^5^Génomique Métabolique, Genoscope, Institut François Jacob, CEA, CNRS, Univ Evry, Université Paris-Saclay, 91057 Evry, France*

*.*

* Corresponding authors: Eva Maria Novoa (eva.novoa@crg.eu) and Manolis Kellis (manoli@mit.edu)

**SUPPLEMENTARY FIGURES**

**Figure S1. (A)** Scatter plot of the first four principal component scores of the per-species average RSCU values. Each dot represents a species, and has been colored according to its corresponding domain of life. **(B)** Score density plots of the first four principal components, for each domain of life: Archaea (blue), Bacteria (red), Eukarya (green).

**Figure S2. Codon preferences in *E. coli* as a function of expression levels.** Codon preferences, represented by relative synonymous codon usage (RSCU), are reversed between highly and lowly expressed genes for some amino acids but not for others, as also occurs in S. cerevisiae (Figure 3). Although codon usage varies within a genome, intra-genome differences are small enough that individual sequences still cluster by domain, as seen in Figure 2.


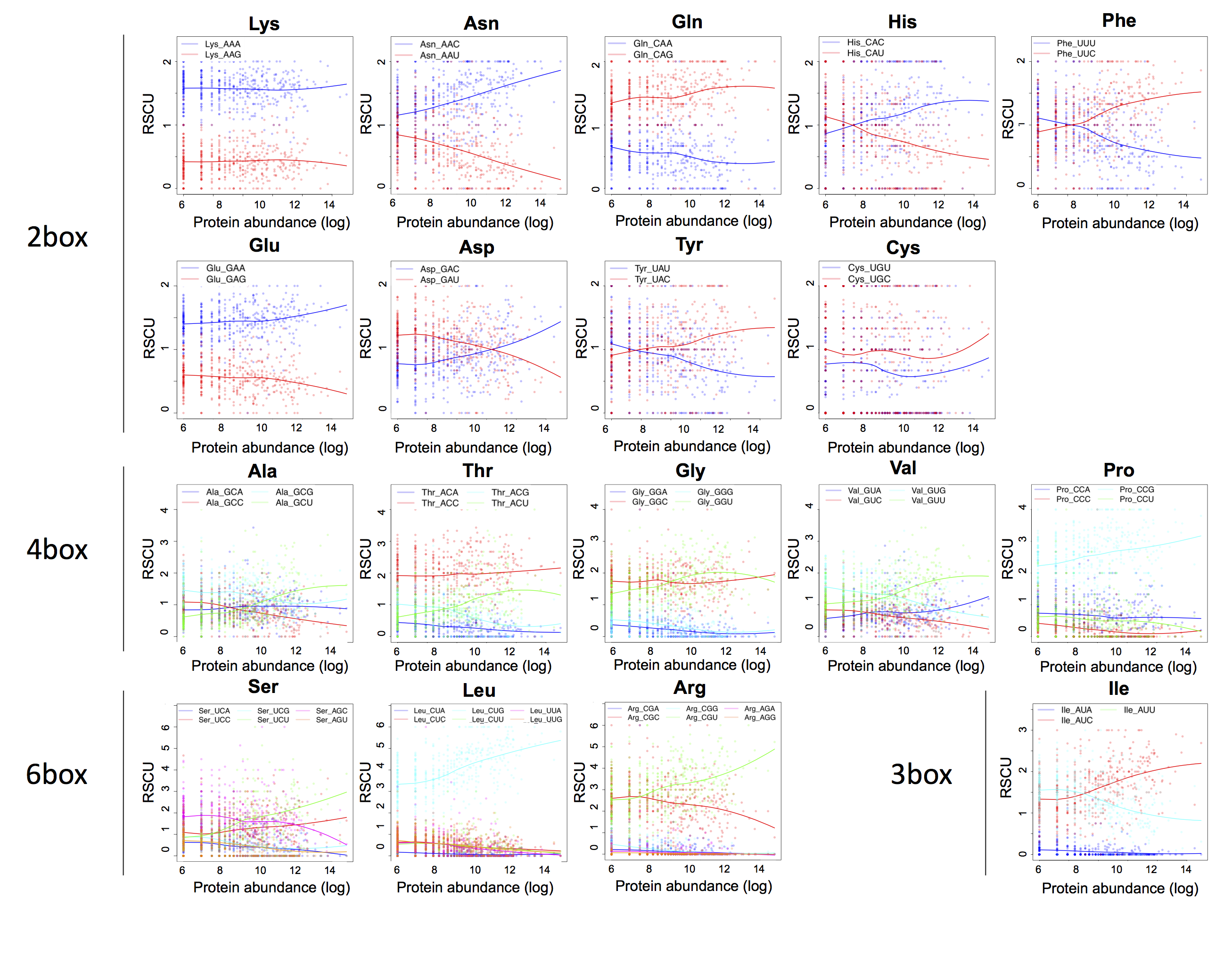


**Figure S3.** ROC curves of the SVM class probabilities, where CDS sequences have been binned based on their sequence length, showing that classification improves with sequence length. CDS sequences longer than 700 nucleotides have not been included in the analysis.

**Figure S4**. Codon covariation measured over all pairs comprised of one codon and the subsequent one encoding for the same amino acid, shown for *E. coli* (**A**) and *P. falciparum* (**B**). Values correspond to standard deviations (SD) from expected. Each codon has been labelled with its corresponding decoding tRNA, following parsimony-extended wobble rules when no Watson-Crick matching tRNA isoacceptor is available. Pairs have been shaded according to the number of standard deviations from expected: dark grey (>+3SD; strongly favoured codon pair), light grey (0-3SD; slightly favoured codon pair), white (<= 0 SD; non-favoured codon pair).

**Figure S5**. Codon covariation for *S. cerevisiae* (**A**) and *E. coli* (**B**) as depicted in Figure S4, highlighting those pairs that are formed by two optimal codons (dark green), two non-optimal codons (red) and codons with intermediate optimality (yellow). Optimal and non-optimal codons have been defined as those that are highly abundant and lowly abundant in highly expressed proteins, and their relative abundance is shown for each individual amino acid and codon.

**Figure S6**. Scatter plot of the first two principal components (left panel), and second and third components (right panel) of the scores of the matrix of RSCPU values of the 1675 EMBLCDS species used in this work show that species belonging to different domains largely overlap. Each dot represents a species, and has been colored according to its corresponding domain.

**Figure S7. (A)** Variance in translation speed between codons that encode for the same amino acid, using the data from Chevance et al. for a bacterial system ^1^ and the data from Gardin et al. for an eukaryotic system ^2^. Amino acids have been sorted according to their variance in Bacteria, and normalized to the amino acid with highest variance, for each domain of life separately. (**B**) Schematic representation of the pairing of the Shine-Dalgarno sequence upstream the start codon with the 16s rRNA, promoting ribosome recruitment. **(C)** Representation of the tRNA:codon pairings that occur for decoding arginine codons in Archaea and Bacteria. In the upper panels, the average relative synonymous codon usage (RSCU) values for each arginine codon are depicted. In the bottom panels, the average relative gene frequencies (RGF) for each tRNA arginine isodecoder are depicted. In Archaea, tRNA^Arg^(ACG) isoacceptors do not exist. In Bacteria, tRNA^Arg^(ACG) is converted to tRNA^Arg^(ICG) by tRNA adenosine deaminases (tadA), which can then decode CGC, CGU and CGA codons. It is worth noticing that inosine preferentially pairs with C and U (i.e. CGC and CGU codons in the case of arginine). The appearance of tadA enzymes in Bacteria might be responsible for the preferred usage of CGC and CGU arginine codons in Bacteria, whereas the lack of this enzyme in Archaea might explain why AGA and AGG codons are preferentially used in this domain.

**SUPPLEMENTARY TABLES**

**Table S1. Contribution of individual codons to the separation of species in Principal Component Analysis.** Loadings of the first three PCs are shown, and are ranked by their PC2 loadings.

| **Codon** | **PC1_loadings** | **PC2_loadings** | **PC3_loadings** |
| --- | --- | --- | --- |
| AGA | -0.249570249 | -0.430620967 | -0.308800612 |
| AGG | -0.045089501 | -0.368229493 | -0.017614132 |
| AUA | -0.079591058 | -0.199739913 | -0.089729245 |
| CUC | 0.142797731 | -0.180531532 | 0.428000402 |
| GGA | -0.111261817 | -0.149504743 | -4.02E-05 |
| GAG | 0.084287541 | -0.118422823 | 0.085352025 |
| AAG | 0.106692493 | -0.113321544 | 0.063769116 |
| UCC | 0.115340853 | -0.106778215 | 0.017997777 |
| UAC | 0.094389509 | -0.097178512 | 0.096146212 |
| CCC | 0.099456689 | -0.097130865 | 0.027819046 |
| CAG | 0.116659961 | -0.095530268 | -0.064238296 |
| CUU | -0.089699208 | -0.087330284 | 0.014704243 |
| CAC | 0.086513518 | -0.081665025 | 0.088375712 |
| GAC | 0.103572729 | -0.078244599 | 0.065620788 |
| UUC | 0.119939399 | -0.07796314 | 0.084748046 |
| AAC | 0.106388974 | -0.068280617 | 0.05716879 |
| GGG | 0.027694809 | -0.058325169 | 0.063347592 |
| ACA | -0.136122475 | -0.052630517 | 0.014748339 |
| UGC | 0.104234536 | -0.04187563 | -0.041592026 |
| GCA | -0.121510204 | -0.030024194 | -0.020683019 |
| GUA | -0.110091695 | -0.029485893 | -0.084887471 |
| GUC | 0.127709463 | -0.02863166 | 0.219491655 |
| GCC | 0.14197898 | -0.027512629 | -0.071857439 |
| CCU | -0.128473506 | -0.019912885 | -0.015574748 |
| CGG | 0.13721217 | -0.019619548 | 0.086126206 |
| CUA | -0.055252863 | -0.019237635 | 0.061968769 |
| ACU | -0.123363679 | -0.012359123 | -0.007713101 |
| GCU | -0.131235979 | -0.007451539 | 0.030347605 |
| AUG | 0 | 0 | 1.06E-22 |
| UAA | 0 | 0 | 0 |
| UAG | 0 | 0 | 0 |
| UGA | 0 | 0 | 0 |
| UGG | 0 | 0 | 0 |
| UCU | -0.131746787 | 0.001880191 | 0.022769336 |
| AUC | 0.19674759 | 0.003853312 | 0.070874985 |
| UCA | -0.128208354 | 0.004114436 | 0.030400335 |
| GUU | -0.142956541 | 0.015657127 | 0.00854657 |
| UCG | 0.146287445 | 0.016254101 | 0.136035055 |
| AGC | 0.109136614 | 0.024428574 | -0.191582573 |
| CCA | -0.138460222 | 0.026792591 | 0.084344645 |
| ACC | 0.165689523 | 0.0272038 | -0.153257374 |
| ACG | 0.09382521 | 0.037740803 | 0.146580129 |
| UGU | -0.104234536 | 0.04187563 | 0.041592026 |
| GUG | 0.125345088 | 0.042301676 | -0.143079823 |
| AGU | -0.110804488 | 0.060120723 | -0.015637862 |
| GCG | 0.110738841 | 0.06518781 | 0.062111412 |
| CUG | 0.293319307 | 0.065765356 | -0.528329437 |
| AAU | -0.106388974 | 0.068280617 | -0.05716879 |
| GGC | 0.182227617 | 0.075985135 | -0.116177658 |
| UUU | -0.119939399 | 0.07796314 | -0.084748046 |
| GAU | -0.103572729 | 0.078244599 | -0.065620788 |
| CAU | -0.086513518 | 0.081665025 | -0.088375712 |
| CGA | -0.026135158 | 0.082407915 | 0.195551981 |
| CCG | 0.167434109 | 0.090115232 | -0.096481605 |
| CAA | -0.116659961 | 0.095530268 | 0.064238296 |
| UAU | -0.094389509 | 0.097178512 | -0.096146212 |
| UUG | -0.049862613 | 0.108166663 | 0.064229138 |
| UUA | -0.241314166 | 0.113199547 | -0.04085649 |
| AAA | -0.106692493 | 0.113321544 | -0.063769116 |
| GAA | -0.084287541 | 0.118422823 | -0.085352025 |
| GGU | -0.098689217 | 0.131697262 | 0.052794006 |
| AUU | -0.11715599 | 0.195783835 | 0.018763531 |
| CGC | 0.248879485 | 0.285658183 | -0.131769571 |
| CGU | -0.065331824 | 0.450412939 | 0.176560485 |

**Table S2. Number of sequences included in the analysis for each domain and phylum**

| **Archaea** |  | **344976** |
| --- | --- | --- |
|  | Crenarchaeota | 96570 |
|  | Euryarchaeota | 244541 |
|  | Thaumarchaeota | 3865 |
| **Bacteria** |  | **12494081** |
|  | Actinobacteria | 1463504 |
|  | Aquificae | 21808 |
|  | Armatimonadetes | 2876 |
|  | Bacteroidetes-Chlorobigroup | 11623 |
|  | Caldiserica | 3164 |
|  | Chlamydiae-Verrucomicrobia | 22314 |
|  | Chloroflexi | 43837 |
|  | Cyanobacteria | 188083 |
|  | Deferribacteres | 16656 |
|  | Deinococcus | 83977 |
|  | Dictyoglomi | 7356 |
|  | Elusimicrobia | 5935 |
|  | Fibrobacteres-Acidobacteria | 29792 |
|  | Firmicutes | 3648592 |
|  | Fusobacteria | 74262 |
|  | Gemmatimonadetes | 3935 |
|  | Nitrospirae | 2038 |
|  | Planctomycetes | 50758 |
|  | Proteobacteria | 6386942 |
|  | Spirochaetes | 133331 |
|  | Synergistetes | 43601 |
|  | Tenericutes | 168253 |
|  | Thermodesulfobacteria | 7199 |
|  | Thermotogae | 74245 |
| **Eukarya** |  | **1300908** |
|  | Alveolata | 127650 |
|  | Amoebozoa | 57373 |
|  | Cryptophyta | 25708 |
|  | Euglenozoa | 42672 |
|  | Haptophyceae | 43883 |
|  | Opisthokonta | 758248 |
|  | Rhodophyta | 6157 |
|  | Stramenopiles | 62961 |
|  | Viridiplantae | 176256 |

**Table S3. Accuracy of phylum prediction**

| **Domain** | **Phylum** | **Accuracy** |
| --- | --- | --- |
| ARCHAEA |  |  |
|  |  |  |
|  | **Global** | **0.901** |
|  | Crenarchaeota | 0.905 |
|  | Euryarchaeota | 0.898 |
|  |  |  |
| BACTERIA |  |  |
|  | **Global** | **0.359** |
|  | Actinobacteria | 0.622 |
|  | Bacteroidetes | 0.785 |
|  | Chlamydiae | 0.899 |
|  | Chloroflexi | 0.523 |
|  | Cyanobacteria | 0.585 |
|  | Deinococcus | 0.566 |
|  | Fibrobacteres | 0.667 |
|  | Firmicutes | 0.357 |
|  | Fusobacteria | 0.838 |
|  | Planctomycetes | 0.106 |
|  | Proteobacteria | 0.434 |
|  | Spirochaetes | 0.293 |
|  | Synergistetes | 0.258 |
|  | Tenericutes | 0.553 |
|  | Thermotogae | 0.598 |
|  |  |  |
| EUKARYA |  |  |
|  | **Global** | **0.582** |
|  | Alveolata | 0.431 |
|  | Amoebozoa | 0.774 |
|  | Cryptophyta | 0.811 |
|  | Euglenozoa | 0.810 |
|  | Haptophyceae | 0.768 |
|  | Opisthokonta | 0.570 |
|  | Rhodophyta | 0.761 |
|  | Stramenopiles | 0.458 |
|  | Viridiplantae | 0.577 |

**Table S4. Performance of phylum prediction**

| **Domain** | **Phylum** | **Sensitivity** | **Specificity** | **posPredValue** | **negPredValue** |
| --- | --- | --- | --- | --- | --- |
| ARCHAEA |  |  |  |  |  |
|  | Crenarchaeota | 0.901 | 0.901 | 0.782 | 0.958 |
|  | Euryarchaeota | 0.901 | 0.901 | 0.958 | 0.782 |
| BACTERIA |  |  |  |  |  |
|  | Actinobacteria | 0.622 | 0.974 | 0.699 | 0.964 |
|  | Bacteroidetes | 0.785 | 0.975 | 0.022 | 1.000 |
|  | Chlamydiae | 0.899 | 0.981 | 0.060 | 1.000 |
|  | Chloroflexi | 0.523 | 0.948 | 0.026 | 0.999 |
|  | Cyanobacteria | 0.585 | 0.967 | 0.167 | 0.995 |
|  | Deinococcus | 0.566 | 0.981 | 0.134 | 0.998 |
|  | Fibrobacteres | 0.667 | 0.959 | 0.029 | 0.999 |
|  | Firmicutes | 0.357 | 0.847 | 0.401 | 0.821 |
|  | Fusobacteria | 0.838 | 0.958 | 0.083 | 0.999 |
|  | Planctomycetes | 0.106 | 0.926 | 0.319 | 0.759 |
|  | Proteobacteria | 0.434 | 0.888 | 0.711 | 0.712 |
|  | Spirochaetes | 0.293 | 0.952 | 0.048 | 0.994 |
|  | Synergistetes | 0.258 | 0.977 | 0.029 | 0.998 |
|  | Tenericutes | 0.553 | 0.956 | 0.117 | 0.995 |
|  | Thermotogae | 0.598 | 0.971 | 0.086 | 0.998 |
| EUKARYA |  |  |  |  |  |
|  | Alveolata | 0.431 | 0.976 | 0.650 | 0.942 |
|  | Amoebozoa | 0.774 | 0.976 | 0.605 | 0.989 |
|  | Cryptophyta | 0.811 | 0.959 | 0.289 | 0.996 |
|  | Euglenozoa | 0.810 | 0.951 | 0.356 | 0.993 |
|  | Haptophyceae | 0.768 | 0.975 | 0.520 | 0.992 |
|  | Opisthokonta | 0.570 | 0.854 | 0.846 | 0.585 |
|  | Rhodophyta | 0.761 | 0.959 | 0.084 | 0.999 |
|  | Stramenopiles | 0.458 | 0.922 | 0.233 | 0.970 |
|  | Viridiplantae | 0.577 | 0.901 | 0.474 | 0.933 |

**SUPPLEMENTARY REFERENCES**

1 Chevance, F. F., Le Guyon, S. & Hughes, K. T. The effects of codon context on in vivo translation speed. *PLoS Genet* **10**, e1004392, doi:10.1371/journal.pgen.1004392 (2014).

2 Gardin, J. *et al.* Measurement of average decoding rates of the 61 sense codons in vivo. *Elife* **3**, doi:10.7554/eLife.03735 (2014).
